# Influence of Heat Treatment on Tea Polyphenols and Their Impact on Improving Heat Tolerance in *Drosophila melanogaster*

**DOI:** 10.3390/foods12203874

**Published:** 2023-10-23

**Authors:** Jianfeng Huang, Xinxin Su, Qiyan Jia, Haoran Chen, Shaoxiao Zeng, Hui Xu

**Affiliations:** 1 College of Food Science, Fujian Agriculture and Forestry University, Fuzhou 350002, China; hjfeng2023@163.com (J.H.); goeunsu@163.com (X.S.); zuzana0815@163.com (Q.J.); chenhaoran6054@163.com (H.C.); zsxfst@163.com (S.Z.); 2 Fujian Provincial Key Laboratory of Quality Science and Processing Technology in Special Starch, Fujian Agriculture and Forestry University, Fuzhou 350002, China

**Keywords:** tea polyphenols, *Drosophila melanogaster*, heat tolerance, transcriptomics, metabolomics

## Abstract

This study investigated the potential mechanism of action of tea polyphenols (TPs), one of the major active ingredients in tea, to enhance heat resistance in Drosophila and the attenuating effect of heat treatment of TPs on their efficacy. The results showed that TPs were able to prolong the average survival time of Drosophila under high-temperature stress (*p* < 0.05), but the effect of TPs in prolonging the survival time of *Drosophila melanogaster* was significantly reduced (*p* < 0.05) with increasing TP heat-treatment time until it disappeared. The composition of TPs changed after heat treatment. It was also shown that the weakening of the effect of TPs in improving the heat tolerance of Drosophila was related to the decrease in the content of catechins and phenolic acids in their fractions as well as with the increase in the content of laccase. Transcriptomic analysis showed that the effect of TPs on heat tolerance in *Drosophila melanogaster* was closely related to the longevity regulation pathway, the neuroactive ligand–receptor interaction signaling pathway, and the drug metabolism–cytochrome P450 pathway. Metabolomics analysis showed that the effect of TP intervention in improving the body’s heat tolerance was mainly related to amino acid metabolism and energy metabolism. However, thermal processing weakened the relevance of these transcriptomes and metabolomes. The present study reveals the mechanism of action by which heat-treated TPs affect the body’s heat tolerance, which is important for the development and utilization of the heat-protection function of tea.

## 1. Introduction

With the increase in the global population, the acceleration of urbanization, the rapid consumption of energy, and the sharp increase in carbon emissions [1] (Sher et al., 2021), the greenhouse effect has continued to intensify, leading to a continuous rise in the global average temperature and the increasing frequency of extreme hot weather in some regions of the planet, which not only affects the living environment of humans but also threatens their physical health. Improving the heat resistance of the human body to cope with high-temperature environments has received widespread attention, and natural foods and beverages have become the best choice for people.

In southern China, tea has been used since ancient times as a daily beverage to protect against summer heat. Previous studies have shown that tea increases the body’s heat resistance. Ref. [2] (Huang Yan et al., 2020) found that aqueous extracts of green tea, white tea, oolong tea, and black tea could improve the heat tolerance of female Drosophila, but the mechanism of action was not studied in depth. TPs is a general term for polyphenol compounds in tea [3] (Zhao & Zhang, 2020), which are among the main active ingredients in tea, with physiological activities such as antioxidant, antitumor, intestinal flora-regulating, and bacteriostatic activities. TPs have been developed and researched in the fields of food, medicine, and daily doses of chemicals [4] (Li et al., 2023), affecting the flavor quality and health functions of tea. Thermal processing is an essential step in tea processing, including the green-tea killing process, roasting of oolong tea, and drying of various types of tea. Tea at high temperatures accelerates the oxidation, sublimation, and other reactions of the active ingredient, TPs, thus affecting the content and composition of TPs [5] (Qiusheng et al., 2014), which in turn inevitably affects their function. Therefore, it is of great scientific value to investigate whether TPs after thermal processing have the effect of increasing the heat resistance of the organism and to study the molecular mechanism of their action.

In this study, we took TPs, among the major active ingredients in tea, as the research object, simulated the roasting process in tea processing to heat treat TPs (140 °C), and then added TPs and heat-treated TPs into the culture medium to feed *Drosophila melanogaster* for 3 days. We observed the survival time of *Drosophila* at high temperatures (36 ± 1 °C) and analyzed the mechanism of TPs’ action to improve the heat-tolerance properties of Drosophila at both the gene level and the metabolic level, with the aim of providing a scientific basis for the further exploitation and utilization of tea.

## 2. Materials and Methods

### 2.1. Reagents and Materials

Strain W1118 wild-type *Drosophila melanogaster* with red eyes were provided by the Third Institute of Oceanography (Xiamen, China), Ministry of Natural Resources. Rearing conditions: temperature: (25 ± 1) °C, humidity: (50 ± 5%), light:dark = 12 h:12 h.

Other materials included ether (Sinopharm Group Chemical Reagent Co., Ltd., Shanghai, China), propionic acid (Sinopharm Group Chemical Reagent Co., Ltd.), TPs (Shanghai Yi En Chemical Technology Co., Ltd., Shanghai, China), acetonitrile Merck (Darmstadt, Germany), methanol Merck (Darmstadt, Germany), formic acid CNW (Shanghai, China), 2-chloro phenylalanine GL Biochem Ltd. (Shanghai, China), RNA 2100 assay reagent (Agilent, Santa Clara, CA, USA), agarose (agarose gel reagent) (Biowest, Nuaillé, France), ammonium acetate (Sigma, Burlington, MA, USA).

### 2.2. TP Concentration Screening Experiment

Referring to the method of [6] (HUANG Yan et al., 2020), the basal medium was prepared by adding 68 g of cornstarch to 230 mL of distilled water and mixing well to form a sticky paste. Then, 52 g of sucrose and 10 g of agar powder were added to 150 mL of distilled water and stirred and mixed well to dissolve completely. Then, the two solutions were combined, and 10 g of yeast and 3.2 mL of propionic acid were added to fully adjust the mixture, which was then dispensed into 125 mL wide-mouth bottles, with 35 g of medium in each bottle.

Then, 0.022 g, 0.044 g, 0.088 g, and 0.176 g of TPs were added to 35 g of basal medium to obtain 0.0625%, 0.1250%, 0.2500%, and 0.5000% of TP experimental medium in different dose groups.

Female *Drosophila melanogaster* that had fledged and not mated within 8–10 h were cultured in basal medium for 3 days. After that, the female *Drosophila melanogaster* were transferred into basal medium (blank control group) and TP experimental medium (0.0625% concentration group, 0.1250% concentration group, 0.2500% concentration group, 0.5000% concentration group) for another 3 days. The results are shown in the following table. On the 7th d, Drosophila were transferred to empty bottles and placed under an artificial climatic chamber (Ningbo Jiangnan Instrument Factory, Ningbo, China) for thermal exposure (temperature set at 36 ± 1 °C and humidity at 50 ± 5%), and the number of Drosophila deaths was observed and recorded every 0.5 h until all of them died. The lethal time of 50% (LT_50_), mean survival time (T), and maximum survival time (T_max_) were used to evaluate the heat resistance of Drosophila.

### 2.3. Effect of TP on the Heat Resistance of Drosophila

The experimental groups were set as 4 groups. For each group, 10 g of TPs was weighed, placed on kraft paper, and put into a blower drying oven. The temperature was set at 140 degrees Celsius, and the times were set as 0 h, 2 h, 4 h, and 6 h, respectively. Referring to the method of medium preparation in Section 2.2, the medium for high-temperature treatment of TPs for 0 h, 2 h, 4 h, and 6 h was prepared in accordance with the concentration of TPs of 0.2500% as screened out and by the same procedure as that in Section 2.2. The Drosophila heat-exposure experiment was carried out with high-temperature-treated TPs in the same steps as in the heat exposure experiment in Section 2.2.

### 2.4. Analysis of TP Composition

The same method as in Section 2.3 was used to prepare high-temperature-treated TPs. Fifty milligrams of the sample was placed in a 2.0 mL centrifuge tube, 800 μL of 80% methanol was added, and a grinder (Ningbo Xinzhi Bio-technology Co., Ltd., Ningbo, China) was applied to grind the sample at 65 Hz for 90 s. The sample was vortexed and shaken to mix thoroughly and then sonicated at 4 °C for 30 min. Centrifugation was carried out at 4 °C for 15 min at 12,000 rpm, and then the tube was allowed to stand at −20 °C for 1 h. Then, 5 microliters of the internal standard (0.14 mg/mL DCP) was added to the supernatant, and 200 μL of the supernatant was transferred to the injection vial. High-resolution mass spectrometry (Thermo Fisher Scientific, Waltham, MA, USA) was applied to carry out chromatographic analysis and mass spectrometry detection with reference to the mass spectrometry detection parameters (Supplementary Table S1).

### 2.5. Transcriptomic Sequencing

Similar to the experimental steps in Section 2.2 and Section 2.3 to culture the experimental Drosophila, according to the screened TP concentration of 0.2500%, the experiment was set up with a blank control group (Group C), TP intervention group (TP group), 6 h high-temperature treatment (140 °C) TP intervention group (TP6 group). Group C, the TP group, and the TP6 group were subjected to heat exposure in an artificial climatic chamber at 36 ± 1 °C. Groups C_H, TP_H and TP6_H were obtained after 2 h of heat exposure. The Drosophila of each group were transferred into empty bottles, and the surviving Drosophila were removed and anesthetized with ether. One hundred grams was randomly selected, placed in bottles wrapped in tin foil, filled with liquid nitrogen, and frozen at −80 °C for spare use. Sample RNA was extracted by applying the TRIzol method, and the cDNA library was analyzed by using sequencing-by-synthesis technology with the Illumina HiSeq high-throughput sequencing platform [7] (Hidehiro et al., 2017). The obtained high-quality reads were then analyzed bioinformatically using a 2100 Bioanalyzer (Agilent), and functional annotation was carried out by comparing the unigenes with the GO and KEGG databases [8] (Luo et al., 2017).

### 2.6. Metabolomics Experiments

Drosophila from groups C, TP, TP6, C_H, TP_H, TP6_H were transferred into empty flasks. The surviving females were taken out, anaesthetised with ether, and 100 g of females from each group were randomly selected to be wrapped in tin foil and filled with liquid nitrogen, and then frozen at −80 °C for spare use. After the samples were thawed slowly at 4 °C, an appropriate amount of sample was added to precooled methanol:acetonitrile:water (2:2:1) solution by vortex mixing, followed by low-temperature ultrasound for 30 min, static at −20 °C for 10 min, and centrifugation at 14,000× *g* and 4 °C for 20 min. The supernatant was taken and vacuum dried. Mass spectrometry analysis (AB SCIEX) was performed by adding 100 μL of acetonitrile aqueous solution (acetonitrile: water = 1:1, *v*/*v*) to redissolve, vortex, and centrifuge at 14,000× *g* at 4 °C for 15 min, and the supernatant was added to the sample for analysis. The metabolite results were analyzed by chromatographic conditions, Q-TOF mass spectrometry conditions (Supplementary Tables S2 and S3), and OPLS-DA model calculations using the R (3.3.2) ropls package. The reliable differential metabolites analyzed in this experiment were screened according to the method in [9] (Zhao et al., 2019). The identified metabolites were annotated using the KEGG database and functionally classified by differential electrochemical mass spectrometry (DEMS). The KEGG pathways were analyzed for significant enrichment of differential metabolites, and the pathways with significant enrichment of differential metabolites were obtained to identify the metabolic pathways and signal transduction pathways in which the differential metabolites were mainly involved.

### 2.7. Data Analysis

The experimental data were statistically analyzed using IBM SPSS statistics 26.0 for one-way ANOVA, and Tukey’s multiple comparison method was used to statistically analyze the experimental data. The data were statistically analyzed with *p* < 0.05 indicating significant differences. Data are expressed as the mean ± standard error (mean ± SEM) and graphically presented using GraphPad Prism 9.1.1. (GraphPad Software Inc., La Jolla, CA, USA).

## 3. Results and Discussion

### 3.1. Effect of Different Concentrations of TP on the Heat Tolerance of Drosophila melanogaster

**Figure 1:**
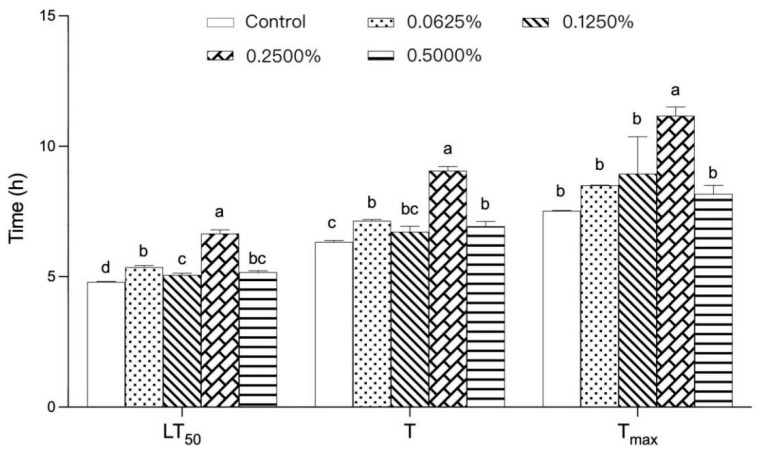
Effect of TPs on the lifespan of heat-exposed female *Drosophila melanogaster*. Note: The letters a, b, c, and d are used as symbols for the analysis of variance, with variance between representations with different letters and no variance between representations with the same letter.

*Drosophila melanogaster* is a model organism commonly used in various biomedical studies, and it has been noted that Drosophila and human genes share more than 60% similarity [10] (Vonesch et al., 2016). As a thermotropic animal, Drosophila has temperature sensors capable of sensing very small temperature changes [11] (Gallio et al., 2011), and female Drosophila are less sensitive to heat than males and can be observed for longer periods of time compared with males under heat-treatment tests [2,12] (Huang et al., 2022; Parkash et al., 2014). Therefore, female *Drosophila melanogaster* was chosen as the model in this study. As shown in Figure 1, in the heat-exposure experiment, the lifespan of Drosophila ingesting different concentrations of TPs at high temperatures was significantly higher than that of the control group (*p* < 0.05). With the increase in TP concentration, LT50, T, and Tmax all showed an overall trend of increasing and then decreasing. The LT50, T, and Tmax of Drosophila reached their maximum values at the 0.2500% TP concentration (*p* < 0.05), which were 1.27, 1.43, and 1.48 times higher than those of the control group, respectively. This suggests that the effect of TPs on Drosophila heat tolerance may be related to their dose, and the concentration of 0.2500% has the best effect on the heat tolerance of Drosophila. Notably, the effect of TPs on the thermotropic action of Drosophila began to diminish when the concentration exceeded 0.2500%, and it is worthwhile to further explore the reasons for this.

### 3.2. Effect of TPs on the Thermal Tolerance of Drosophila after High-Temperature Treatment

**Figure 2:**
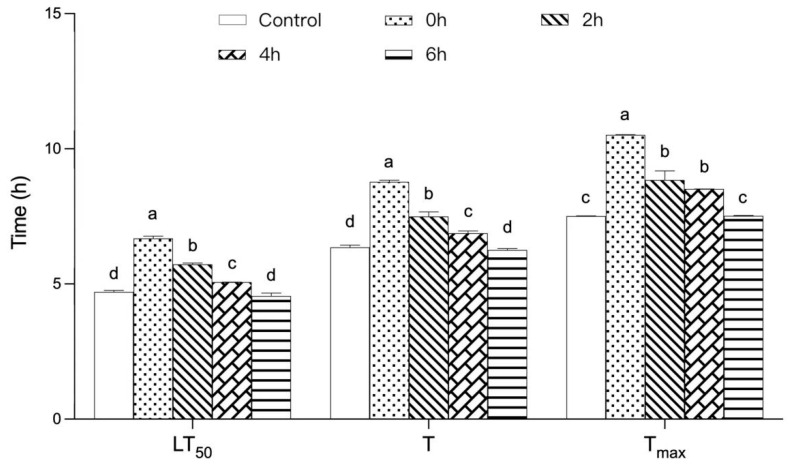
Effect of TPs with different heat-treatment times on the lifespan of heat-exposed female *Drosophila melanogaster*. Note: The letters a, b, c, and d are used as symbols for the analysis of variance, with variance between representations with different letters and no variance between representations with the same letter.

As shown in Figure 2, the LT50, T, and Tmax of Drosophila showed a significant decreasing trend (*p* < 0.05) with increasing high-temperature treatment time. Compared with the 0 h group, the LT50, T, and Tmax of Drosophila were reduced by 32%, 29%, and 29%, respectively, after 6 h of high-temperature treatment. This may be because high temperature reduced the relative content of polyphenols, the antioxidant effect exerted by TPs was reduced, the reaction of oxygen radicals in the organism was reduced, and the ability of scavenging hydroxyl radicals (-OH) and organic radicals (DPPH-) was weakened with the increase in the time of high-temperature treatment [13] (Nan et al., 2021). It is noteworthy that there was no significant change between the blank control group and the 6 h group, suggesting that high-temperature treatment with TPs for 6 h could not improve the heat tolerance of Drosophila. The results showed that the effect of TPs on the heat tolerance of Drosophila weakened with increasing high-temperature treatment time, indicating that the heat-eliminating function of TPs decreased with increasing high-temperature treatment time.

### 3.3. Relative Content Analysis of Polyphenols after TP High-Temperature Treatment

**Figure 3:**
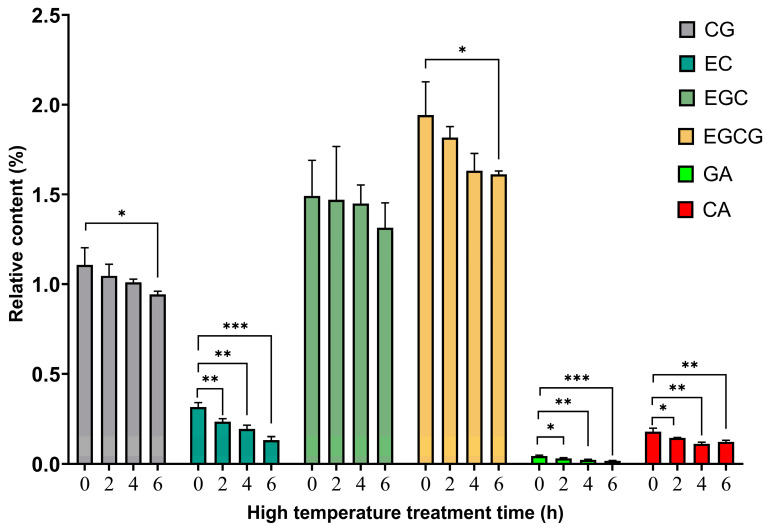
Changes in the relative content of catechin gallate, epicatechin, epigallocatechin, epigallocatechin gallate, gallic acid, and caffeic acid after TP high-temperature treatment. Note: * *p* < 0.05, ** *p* < 0.01, *** *p* < 0.001.

**Figure 4:**
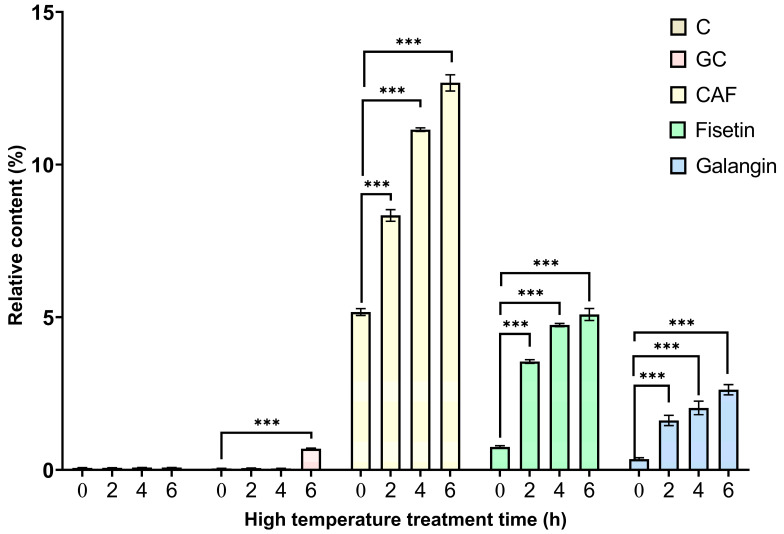
Changes in the relative contents of lacinin, galangin, caffeine, catechin, and gallocatechin after high-temperature treatment of TPs. Note: *** *p* < 0.001.

As seen from Table 1, the relative contents of EGC, CG, and EGCG in TPs before high-temperature treatment of TPs were high, ranging from 1% to 2%, while the contents of C, GC, EC, GA, and CA were low, ranging from 0.01 to 0.40%. It is noteworthy that CAF was the component with the highest relative content before high-temperature treatment, accounting for approximately 5.1471%. As shown in Figure 3, the relative contents of catechins (CG, EC, EGC, EGCG) and phenolic acids (GA, CA) showed a general decreasing trend with increasing high-temperature treatment time. As shown in Figure 4, the relative contents of C and GC showed an overall increasing trend. The results showed that the overall content of polyphenols decreased at high temperatures, which may be because it was oxidized at high temperatures and related to the formation of macromolecular complexes with proteins and other substances [14,15,16,17] (Auger et al., 2010; Dobrini et al., 2020; Huang et al., 2021; Jin-Lian et al., 2008). The increase in GC content with time at high temperatures may be due to isomerization of catechin-like components at high temperatures [18,19,20] (Donlao & Ogawa, 2019; Kothe et al., 2013; Lee et al., 2013). It is worth noting that the contents of CAF, laccasein, and galangin reached highly significant increases (*p* < 0.001) at 2 h, 4 h, and 6 h of high-temperature treatment compared with those before high-temperature treatment, and showed an overall increasing trend with the lengthening of the high-temperature treatment (Figure 4). This indicates that catechins and phenolic acids in the tea were transformed under high temperature, which may promote the formation of CAF and flavonoids. This reveals that heat treatment changed the composition of TPs, thus diminishing the effect of TPs’ action in improving the body’s heat resistance. The above analysis suggests that catechins and phenolic acids may be the main components that exert the effect of TPs to improve the body’s heat resistance, while caffeine, laccase, and galangal may have inhibitory effects on the heat resistance of Drosophila, but further studies are needed.

### 3.4. Transcriptomic Analysis of TPs to Improve Heat Tolerance in Drosophila

**Figure 5:**
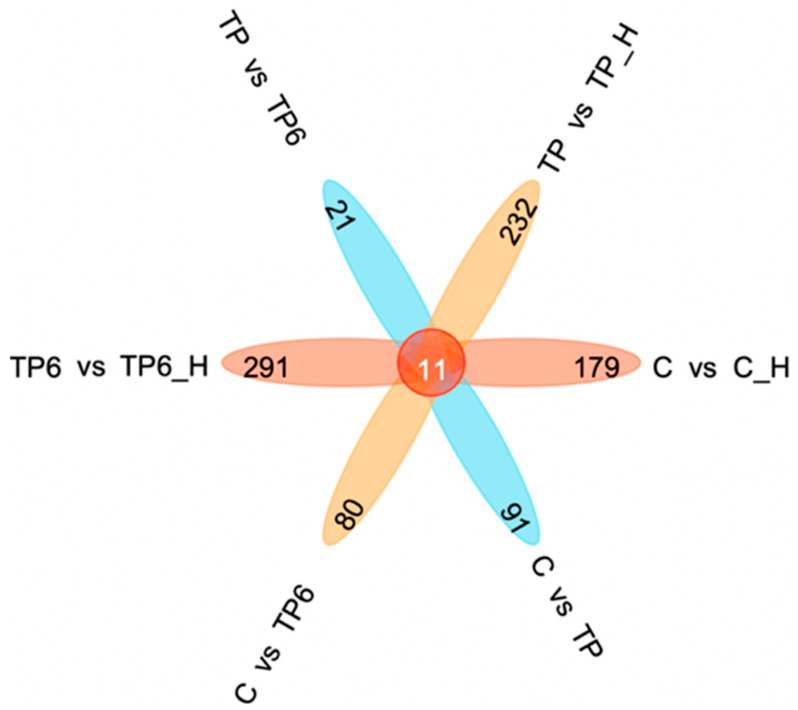
Petal map of differentially expressed genes.

The corresponding C_H, TP_H, and TP6_H groups after high-temperature stress of Drosophila were found to contain 1012, 1403, and 1880 differentially expressed genes, respectively, with a total of 1380 differentially expressed genes identified under C vs. TP, 1725 differentially expressed genes identified under C vs. TP6, and 590 differentially expressed genes identified under TP vs. TP6 (Table 2). In addition, all three groups (C vs. C_H, TP vs. TP_H, TP6 vs. TP6_H) before and after high-temperature stress showed more downregulated genes than upregulated genes; more upregulated genes than downregulated genes were found in the TP group compared with the C group, the TP6 group compared with the C group, and the TP6 group compared with the TP group. The significance of differentially expressed genes was similar in the three groups before and after high-temperature stress, while among the three groups treated with the TP diet (C vs. TP, C vs. TP6, and TP vs. TP6), the significance of differentially expressed genes was the strongest in the C vs. TP group (Figure 5).

The results of GO functional analysis of the differentially expressed genes indicated that the biological processes that were altered in Drosophila after experiencing high-temperature stress were mainly related to monocarboxylic acid biosynthetic processes, protein replication, metabolism, reproduction, and inter-multicellular bioprocesses (Supplementary Table S4). All these biological processes were enriched in five significantly upregulated genes encoding heat-expressed proteins associated with enhanced heat tolerance in Drosophila, namely, Hsp23, Hsp26, Hsp68, Hsp70Ab, and Hsp70Bb (Table 3). Among these genes, Hsp23 and Hsp26 encode small-molecule heat shock proteins, and the rest of the genes are genes encoding heat stress proteins of the 70 family. Hsp23 is considered a key gene affecting heat tolerance in insects, and it has been found that the expression of muscle-specific Hsp23 by *Drosophila melanogaster* promotes protein homeostasis and protects the muscle to reduce the damage caused by heat stress [21] (Kawasaki et al., 2016). Upregulation of Hsp70 expression contributes to increased heat tolerance in Drosophila under high-temperature stress [22] (Stetina et al., 2015). Enhanced heat tolerance in species of the genus Sitophilus has been reported to be associated with high expression of Hsp23, Hsp70, and Hsp90 in third instar larvae [23,24] (Gu et al., 2019; Hu et al., 2014). Heat shock proteins play the most important role in the resistance of organisms to adversity stress, especially high-temperature stress, and their synthesis has been shown to be positively correlated with the heat tolerance of organisms. Therefore, the above results suggest that Drosophila may maintain cellular protein homeostasis at high temperatures by upregulating the expression of the heat shock protein family in vivo to counteract the negative effects of heat stress on proteins and protect the cells from the stresses, thus increasing the organism’s heat tolerance.

Similar to the results of the high-temperature stress group, the main biological processes indicated by the GO of C vs. TP were oxidoreductase activity, exopeptidase activity, metalloexopeptidase activity, and carbohydrate binding (Table 4). All these biological processes were enriched in genes encoding heat stress proteins (Hsp23, Hsp26, Hsp60C, Hsp68, Hsp70Ab, Hsp70Bb) and superoxide dismutase genes (Sod1 and Sod2) (Table 4). Superoxide dismutase, as the main enzyme participating in the antioxidant defense of organisms, plays a role in maintaining the balance of reactive oxygen species content in the body of the organism and prevents cell damage by environmental changes [25] (Yao et al., 2014). It has been suggested that superoxide dismutase increases the level of antioxidant enzymes, thereby prolonging Drosophila lifespan [26,27] (Alscher et al., 2002; Slimen et al., 2014). Therefore, TPs may be able to adapt to high temperatures by synthesizing heat-excited protein genes to maintain normal intracellular functions at high temperatures and by upregulating the expression of Sod genes to regulate antioxidant enzyme activities to counteract the damage caused by high temperatures, thus enhancing heat resistance. In addition, the GO of TP vs. TP6 was significantly enriched in the motor processes of cilia and their related genes (Supplementary Table S5). Cilia play an important role in cell cycle development, cell differentiation, and the cellular stress response [28] (Ringers et al., 2020). Ref. [29] (Bae et al., 2019) found that cilia have an important role in cell survival responses during mitochondrial stress. This suggests that high-temperature-treated TPs may have intervened in the heat stress response in Drosophila by upregulating cilia movement genes.

**Figure 6:**
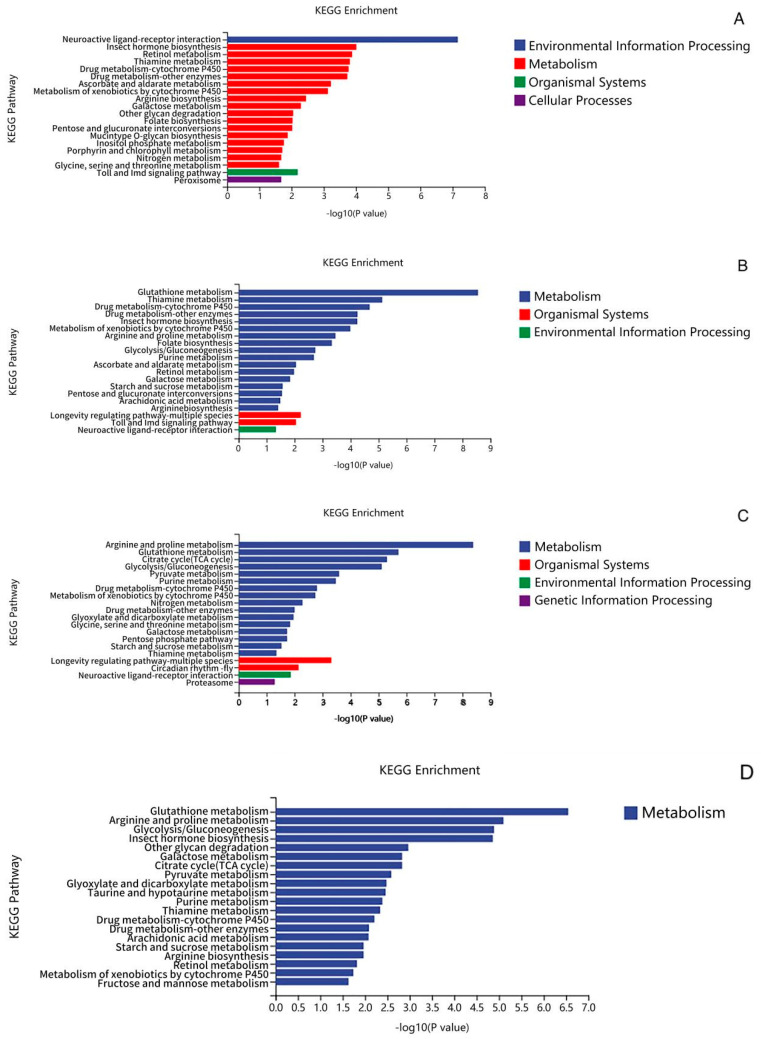
Classification statistics of KEGG pathway enrichment of differentially expressed genes. (**A**): C vs. C_H (**B**): TP vs. TP_H (**C**): TP6 vs. TP6_H (**D**): C vs. TP.

According to KEGG pathway analysis, TPs significantly upregulated the neuroactive ligand–receptor interaction signaling pathway compared with controls (Figure 6B,C). As shown in Table 4, neurotransmitter receptor genes (including Dop1R1, Dop1R2, 5-HT1A, 5-HT2B, GABA-B-R1, GABA-B-R3) were found to be significantly upregulated in the pathway. Among them, mutations carried on dopamine receptor genes (Dop1R1, Dop1R2) have been reported in insect longevity assays; longevity can be extended by signaling through Dop1R2, and the specific dopamine receptor may play an active role in the regulation of longevity in Drosophila [30] (Jiang et al., 2022). The findings of [31] (Li et al., 2019) were similar to the present study in that dietary intervention in Drosophila with black rice anthocyanin extract (BRAE) resulted in a 20% increase in lifespan and delayed the loss of motor function in Drosophila. Their KEGG pathway analyses showed that upregulation of glutathione metabolism, neuroactive ligand–receptor interactions, and upregulation of the genes of these signaling pathways (Zw (Zwischenferment), GstD2, GstE1, Gpx, Gclm, Tsc1, 4E-BP, DopR, and D2R) were closely associated with slowing the aging effects in Drosophila. This suggests that TP may extend the lifespan of Drosophila by modulating the nervous system, regulating the thermal acclimatization state in Drosophila under high-temperature stress, reducing heat stress injury, and increasing heat tolerance.

Notably, upregulated expression of the drug metabolism–cytochrome P450 pathway was found for C vs. C_H, TP vs. TP_H, TP6 vs. TP6_H, and C vs. TP (Figure 6A–D). It has been shown that cytochrome P450, a ubiquitous heme–thiolate protein with potential biotechnological applications, is a key enzyme in drug metabolism and withstands harsh industrial conditions, such as high temperatures, extreme pH, and organic solvents, and has an important impact on both cytokines and thermoregulation, which are required for biotechnological applications [32] (Syed et al., 2014). This suggests that the significant upregulation of cytochrome P450 genes may help to stabilize proteins in Drosophila, thereby mitigating heat stress damage and improving the heat tolerance of Drosophila.

Taken together, the analyses showed that the effect of TPs in improving heat tolerance in Drosophila is closely related to the upregulated expression of the longevity regulatory pathway, neuroactive ligand–receptor interaction signaling pathway, and drug metabolism–cytochrome P450 pathway. High-temperature-treated TPs may affect the heat stress response of Drosophila by interfering with and upregulating the cilia movement process and its related genes.

### 3.5. Metabolomic Analysis of TP for Improving Heat Tolerance in Drosophila

**Figure 7:**
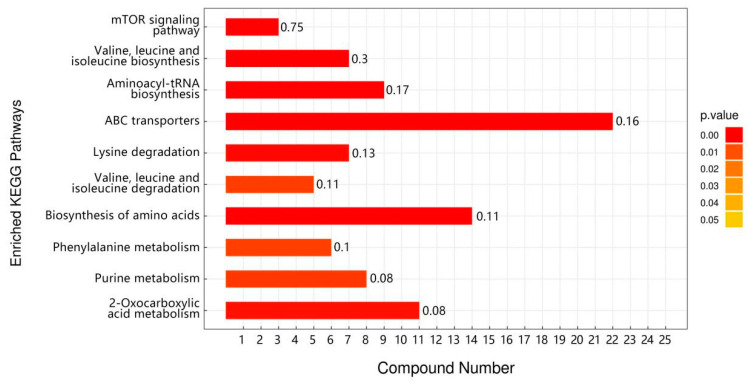
Maps of overlapping KEGG-enriched pathways in the three groups (C vs. C_H, TP vs. TP_H, TP6 vs. TP6_H) before and after high-temperature stress.

There was a total of 51 overlapping differential metabolic markers between the three groups of Drosophila subjected to high-temperature stress (C vs. C_H, TP vs. TP_H, TP6 vs. TP6_H), with the first 20 being observed (Supplementary Table S6). Purine and pyrimidine catabolism as well as valine, leucine, and isoleucine metabolism were also altered in Drosophila under high-temperature stress, according to KEGG enrichment analysis (Figure 7). Ref. [33] (Zhao et al., 2021) found significant changes in the expression levels of genes related to protein and amino acid metabolism in coral-reef fish after heat-stress treatment. The pathways included glycine, serine, and tyrosine metabolism and valine, leucine, and isoleucine metabolism. This suggests that Drosophila’s organismal amino acid metabolism and energy metabolism may be interfered with in response to high temperatures and then rapidly undergo metabolic changes to adapt to its energy supply needs. In addition, there was also a significant enrichment of aminoacyl-tRNA biosynthesis after high-temperature stress (Figure 7). tRNA aminoacylation is an important hallmark of protein synthesis [34] (Robert et al., 1988), which suggests that Drosophila’s protein synthesis capacity may be compromised after heat stress.

**Figure 8:**
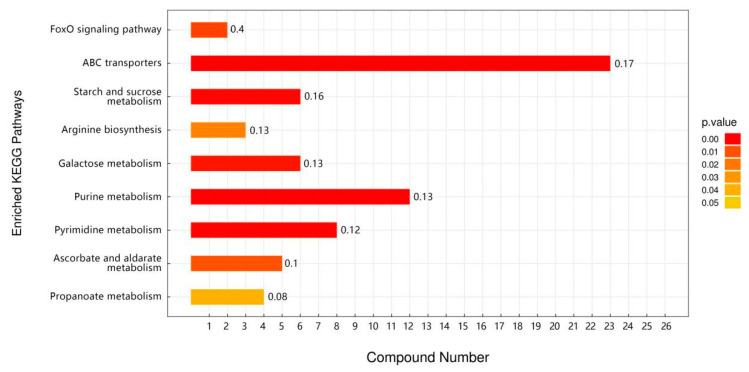
KEGG-enriched pathway maps of the TP intervention group and the control group (C vs. TP).

Based on the differential metabolite and pathway enrichment analyses of C vs. TP, it was found that TP dietary intervention promoted significant upregulation of Drosophila metabolism, increased antioxidant activity, and facilitated the production of heat-stimulating proteins and other related metabolite expression (Supplementary Table S7). This suggests that TPs improve heat tolerance in Drosophila by upregulating the expression of Drosophila heat-tolerance-related metabolites. According to KEGG enrichment analysis, the metabolites in Drosophila after TP intervention were mainly enriched in the pathways of ABC transport, arginine biosynthesis, galactose metabolism, and purine and pyrimidine metabolism (Figure 8). This suggests that TPs may exert a heat-resistant mechanism through the pathways of ABC transport, arginine biosynthesis, galactose metabolism, and purine and pyrimidine metabolism.

**Figure 9:**
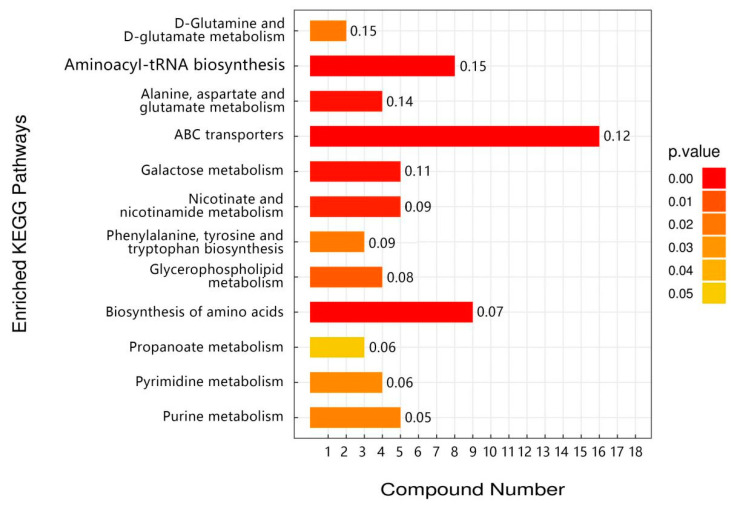
KEGG-enriched pathways in the TP intervention group versus the hyperthermia-treated TP group (TP6 vs. TP).

Based on the differential metabolite and pathway enrichment analyses of TP6 vs. TP, untreated TPs promoted significant upregulation of metabolites related to insect survival and reproduction, amino acid metabolism, neurotransmitters, and purine metabolism (Supplementary Table S7), and the specific enriched pathways were mainly biosynthesis of amino acid metabolism and lipid metabolism (Figure 9). Free amino acids such as phenylalanine, tyrosine, tryptophan, alanine, aspartic acid, and glutamic acid have been reported to be useful in Drosophila for regulating osmotic pressure, detoxifying metabolic wastes from the body, and serving as a source of energy in the body [35] (González et al., 1989). Heat stress leads to lipid deposition, which induces fatty liver and other lipid metabolism disorders and affects lipid metabolism homeostasis [36] (Zhao et al., 2021). Therefore, lipid metabolism plays an important role in maintaining cellular homeostasis under high-temperature stress to alleviate heat-stress-induced lipid metabolism disorders. This suggests that TPs without high-temperature treatment can maintain cellular metabolism by interfering with the body’s amino acid metabolism and energy metabolism and altering the mechanisms of lipid metabolism as a means of coping with heat stress and reducing the accumulation of heat-stress damage, whereas high-temperature-treated TPs may inhibit these mechanisms of action that improve heat tolerance in Drosophila.

In summary, TPs interfere with the accumulation of heat tolerance-related metabolites in the organism, which may be related to amino acid metabolism and energy metabolism, and in this way exert the function of heat elimination. However, high-temperature-treated TPs may have these mechanisms of action inhibited and thus fail to improve heat tolerance in Drosophila.

## 4. Conclusions

In this study, our results suggest that TPs prolong the average survival time of Drosophila under high-temperature stress; however, heat treatment diminishes the effect of TPs in prolonging the survival time of Drosophila. Catechins and phenolic acids may be the main components of TPs, exerting the effect of enhancing heat tolerance in Drosophila. The transcriptomics results indicate that TP enhances heat tolerance in Drosophila by upregulating the expression of genes related to heat stress proteins, the antioxidant protection system, and the thermal stability of the nervous system. Metabolomics results indicate that TPs maintain the normal biological functions of Drosophila under high-temperature stress by activating amino acid metabolism, sugar metabolism, energy metabolism, and other pathways. However, high-temperature treatment weakened the regulatory effects of TPs on the above mechanisms of action. The present study reveals that TPs have a role in enhancing heat tolerance in Drosophila by molecular mechanisms.

## Figures and Tables

**Table 1:** Relative content of polyphenols after TP high-temperature treatment.

Compound Name	Relative Percentage (%)			
	0 h	2 h	4 h	6 h
catechin (C)	0.0611	0.0688	0.0786	0.0763
catechin gallate (CG)	1.1111	1.0343	1.0132	0.9417
epicatechin (EC)	0.3162	0.2337	0.1992	0.1376
epigallocatechin (EGC)	1.5142	1.4809	1.4510	1.3109
epigallocatechin gallate (EGCG)	1.9141	1.7874	1.6762	1.6163
gallocatechin (GC)	0.0498	0.0606	0.0501	0.7000
gallic acid (GA)	0.0440	0.0302	0.0236	0.0173
caffeic acid (CA)	0.1763	0.1488	0.1111	0.1256
caffeine (CAF)	5.1471	8.3875	11.1307	12.5517
laccasein	0.7544	3.5787	4.7548	5.0746
galangal	0.3487	1.6595	2.0904	2.6214

**Table 2:** Statistics on the number of differentially expressed genes.

Control Group vs. Experimental Group	Upregulated	Downregulated	Total
C vs. C_H	358	654	1012
TP vs. TP_H	659	744	1403
TP6 vs. TP6_H	203	1677	1880
C vs. TP	835	545	1380
C vs. TP6	1428	297	1725
TP vs. TP6	540	50	590

**Table 3:** List of overlapping differentially expressed genes in the three groups (C vs. C_H, TP vs. TP_H, TP6 vs. TP6_H) before and after high-temperature stress (partial results).

Gene.ID	Group	log2FC	Regulated
Hsp23	C vs. C_H	10.39543125	up
	TP vs. TP_H	11.29768882	up
	TP6 vs. TP6_H	7.10522365	up
Hsp26	C vs. C_H	6.179345334	up
	TP vs. TP_H	7.037563739	up
	TP6 vs. TP6_H	5.041077323	up
Hsp68	C vs. C_H	5.359082364	up
	TP vs. TP_H	4.551601646	up
	TP6 vs. TP6_H	4.133381398	up
Hsp70Ab	C vs. C_H	3.565616719	up
	TP vs. TP_H	5.622981232	up
	TP6 vs. TP6_H	3.252050367	up
Hsp70Bb	C vs. C_H	4.044325616	up
	TP vs. TP_H	3.265791372	up
	TP6 vs. TP6_H	2.048701979	up

Note: Gene.ID: gene ID; Group: control group vs. experimental group; log2FC: logarithmic value of the fold difference in expression vs. 2; up: upregulation of expressed genes in the experimental group compared with the control group, the same as below.

**Table 4:** List of differentially expressed genes in the TP intervention and control groups (C vs. TP) (partial results).

Gene.ID	log2FC	Regulated	NR_Annotation
Hsp23	10.39543125	up	heat shock protein 23
Hsp26	6.710522365	up	heat shock protein 26
Hsp60C	4.111382603	up	heat shock protein 60C
Hsp68	6.263508306	up	heat shock protein 68
Hsp70Bb	5.252050367	up	heat shock protein 70Bb
Hsp70Ab	5.359082364	up	heat shock protein 70Ab
Sod1	2.367690793	up	Superoxide dismutase 1
Sod2	2.643104457	up	Superoxide dismutase 2
Dop1R1	2.403899212	up	dopamine receptor gene R1
Dop1R2	2.739719490	up	dopamine receptor gene R2
GABA-B-R1	3.714866365	up	g-aminobutyric acid (GABA) type B receptor subunit 1
GABA-B-R3	2.085191171	up	g-aminobutyric acid (GABA) type B receptor subunit 3
5-HT1A	2.33974916	up	5-hydroxytryptamine-1A
5-HT2B	2.51624293	up	5-hydroxytryptamine-2B

Note: NR_annotation: NR database annotation results.

## Data Availability

The data used to support the findings of this study can be made available by the corresponding author upon request.

## Supplementary Materials

The following supporting information can be downloaded at: https://www.mdpi.com/article/10.3390/foods12203874/s1.
