# Supplementary material for: Influence of Heat Treatment on Tea Polyphenols and Their Impact on Improving Heat Tolerance in Drosophila melanogaster
Source: Foods. 2023 Oct 23;12(20):3874. doi: 10.3390/foods12203874 (PMC10606210; doi:10.3390/foods12203874)
Supplement: Supplementary file 1 [file foods-12-03874-s001.zip › foods-2648482-supplementary.pdf]

Supplementary Table S1 Mass Spectrometry Detection Parameters

	**positive mode**	**negative mode**
Heater temperature	300 ℃	300 ℃
sheath gas flow rate	45 arb	45 arb
Auxiliary gas flow rate	15 arb	15 arb
exhaust gas flow rate	1 arb	1 arb
Electrospray voltage	3.0 KV	3.2 KV
capillary tube temperature	350 ℃	350 ℃
S-Lens RF Level	30%	60%
scanning mode	Full Scan (m/z 70~1050) with data-dependent Secondary Mass Spectrometry Scan (dd-MS2 , TopN = 10)	
resolution (of a photo)	70000 (primary mass spectrometry (physics)) and 17500 (Secondary Mass Spectrometry	
crash mode	High-energy collisional dissociation（HCD）	

Supplementary Table S2 Chromatographic conditions

	**proceedings**	**explicit condition**
1	Sample injection volume 2 μL, maintain column temperature 25°C at a flow rate of 0.5 mL/min.	
2	Chromatographic mobile phase composition A (A)	water + 25 mmol/L ammonium acetate + 25 mmol/L ammonia
	Chromatographic mobile phase composition B (B)	acetonitrile
3	Gradient elution procedure for chromatographic separations	① 95% chromatographic mobile phase B, elution time 0-0.5 min<br>② B varied linearly from 95 to 65 % with elution times ranging from 0.5 to 7 min<br>③ B varied linearly from 65 to 40 % with an elution time of 7-8 min<br>④ The B concentration was maintained at 40 % and the elution time was 8-9 min<br>⑤ B varied linearly from 40% to 95% with elution times of 9 to 9.1 min<br>⑥ The B concentration was maintained at 95 % and the elution time was 9.1-12 min
4	The sample is placed in a 4°C autosampler throughout the analysis and the samples are analysed continuously in random order	

Supplementary Table S3 ESI Source Conditions

**proceedings**	**prerequisite**
Ion Source Gas1(Gas1)	60
Ion Source Gas2(Gas2)	60
Curtain gas (CUR)	30
source temperature	600°C
IonSapary Voltage Floating (ISVF)	± 5500 V(Positive and negative modes)
TOF MS scan m/z range and accumulation time	60-1000 Da, 0.20 s/spectra,
product ion scan m/z range and accumulation time	25-1000 Da, 0.05 s/spectra
Secondary Mass Spectrometry	information dependent acquisition (IDA), high sensitivity paradigm
Declustering potential (DP)：	± 60 V(Positive and negative modes)
Collision Energy	35 ±15 eV, Exclude isotopes within 4 Da；Candidate ions to monitor per cycle：10

Supplementary Table S4 GO enrichment analysis of differentially expressed genes in Drosophila before and after high temperature stress (partial results)

Control vs Treat	GO.ID	Domain	Term	Up	Down	DEG	Annotated	P value
C vs C_H	GO:0031224	CC	intrinsic component of membrane	85	223	308	3205	3.04E-16
	GO:0016021	CC	integral component of membrane	85	221	306	3179	3.26E-16
	GO:0005576	CC	extracellular region	48	109	157	1307	2.38E-15
	GO:0050830	BP	defense response to Gram-positive bacterium	2	20	22	56	2.53E-12
	GO:0005615	CC	extracellular space	29	69	98	749	8.01E-12
	GO:0044425	CC	membrane part	93	234	327	3716	8.23E-12
	GO:0044421	CC	extracellular region part	39	76	115	966	4.99E-11
	GO:0070011	MF	peptidase activity, acting on L-amino acid peptides	14	65	79	588	6.11E-09
	GO:0008233	MF	peptidase activity	14	66	80	602	8.05E-09
	GO:0008236	MF	serine-type peptidase activity	8	41	49	304	1.98E-08
	GO:0017171	MF	serine hydrolase activity	8	41	49	305	2.21E-08
	GO:0016020	CC	membrane	97	245	342	4201	4.97E-08
	GO:0004252	MF	serine-type endopeptidase activity	8	37	45	279	7.44E-08
	GO:0004175	MF	endopeptidase activity	11	48	59	439	5.58E-07
	GO:0072330	BP	monocarboxylic acid biosynthetic process	7	16	23	109	6.33E-07
	GO:0005887	CC	integral component of plasma membrane	17	42	59	483	1.60E-06
	GO:0019731	BP	antibacterial humoral response	2	14	16	63	2.52E-06
	GO:0016798	MF	hydrolase activity, acting on glycosyl bonds	6	18	24	122	2.61E-06
	GO:0044283	BP	small molecule biosynthetic process	10	31	41	288	3.12E-06
	GO:0046394	BP	carboxylic acid biosynthetic process	8	20	28	163	3.23E-06

Supplementary Table S4 (continued)

Control vs Treat	GO.ID	Domain	Term	Up	Down	DEG	Annotated	P value
TP vs TP_H	GO:0005615	CC	extracellular space	59	108	167	749	5.26E-29
	GO:0005576	CC	extracellular region	110	128	238	1307	1.89E-27
	GO:0044421	CC	extracellular region part	67	116	183	966	8.24E-23
	GO:0045434	BP	negative regulation of female receptivity, post-mating	2	9	11	15	4.61E-09
	GO:0008238	MF	exopeptidase activity	18	13	31	107	7.73E-09
	GO:0046008	BP	regulation of female receptivity, post-mating	2	11	13	22	9.24E-09
	GO:0008237	MF	metallopeptidase activity	23	15	38	152	1.51E-08
	GO:0007621	BP	negative regulation of female receptivity	2	9	11	17	3.50E-08
	GO:0008235	MF	metalloexopeptidase activity	11	10	21	61	7.70E-08
	GO:0070011	MF	peptidase activity, acting on L-amino acid peptides	61	33	94	588	1.79E-07
	GO:0008233	MF	peptidase activity	61	33	94	602	5.39E-07
	GO:0045924	BP	regulation of female receptivity	3	11	14	34	8.88E-07
	GO:0004177	MF	aminopeptidase activity	9	10	19	59	1.04E-06
	GO:0007618	BP	mating	19	19	38	180	1.40E-06
	GO:0030312	CC	external encapsulating structure	14	2	16	46	1.71E-06
	GO:0007320	BP	insemination	0	12	12	28	3.27E-06
	GO:0055085	BP	transmembrane transport	76	44	120	857	3.57E-06
	GO:0042026	BP	protein refolding	0	13	13	33	3.97E-06
	GO:0044706	BP	multi-multicellular organism process	0	12	12	29	5.10E-06
	GO:0007620	BP	copulation	1	12	13	34	5.88E-06
TP6 vs TP6_H	GO:0005615	CC	extracellular space	19	170	189	749	4.43E-23
	GO:0030312	CC	external encapsulating structure	32	0	32	46	3.76E-19
	GO:0044421	CC	extracellular region part	22	186	208	966	1.22E-16
	GO:0042600	CC	chorion	28	0	28	41	1.39E-16
	GO:0005576	CC	extracellular region	37	221	258	1307	1.52E-15
	GO:0097014	CC	ciliary plasm	0	28	28	57	1.58E-11
	GO:0007320	BP	insemination	0	19	19	28	1.70E-11
	GO:0044706	BP	multi-multicellular organism process	0	19	19	29	4.35E-11

Supplementary Table S4 (continued)

Control vs Treat	GO.ID	Domain	Term	Up	Down	DEG	Annotated	P value
	GO:0005929	CC	cilium	1	51	52	163	5.31E-11
	GO:0005930	CC	axoneme	0	27	27	56	6.32E-11
	GO:0046692	BP	sperm competition	0	18	18	27	9.26E-11
	GO:0007620	BP	copulation	1	19	20	34	2.27E-10
	GO:0044441	CC	ciliary part	0	37	37	109	4.80E-09
	GO:0046008	BP	regulation of female receptivity, post-mating	1	13	14	22	2.96E-08
	GO:0003341	BP	cilium movement	0	19	19	39	4.25E-08
	GO:0042026	BP	protein refolding	0	17	17	33	7.70E-08
	GO:0045434	BP	negative regulation of female receptivity, post-mating	1	10	11	15	1.06E-07
	GO:0048029	MF	monosaccharide binding	4	18	22	54	2.24E-07
	GO:0044782	BP	cilium organization	0	40	40	141	4.29E-07
	GO:0004656	MF	procollagen-proline 4-dioxygenase activity	2	12	14	26	5.85E-07

Note：Control vs Treat：Control group vs experimental group；Domain：BP、MF、CC represent biological processes, molecular functions and cellular components, respectively； GO ID： ID of GO term； Term： GO Function； Annotated： Number of all genes annotated to this function； DEG： Differently expressed genes； P value： Enrichment Significance P-value； Up、Down： denote the number of genes up- and down-regulated in Treat compared to Control group, respectively. Same as below。

Supplementary Table S5 GO enrichment analysis of differentially expressed genes in Drosophila with different tea polyphenol dietary interventions (partial results)

Control vs Treat	GO.ID	Domain	Term	Up	Down	DEG	Annotated	P value
C vs TP	GO:0005615	CC	extracellular space	122	62	184	749	5.84E-40
	GO:0005576	CC	extracellular region	147	108	255	1307	4.31E-37
	GO:0044421	CC	extracellular region part	131	71	202	966	3.91E-33
	GO:0050830	BP	defense response to Gram-positive bacterium	3	20	23	56	1.61E-10
	GO:0045434	BP	negative regulation of female receptivity, post-mating	9	2	11	15	3.36E-09
	GO:0046008	BP	regulation of female receptivity, post-mating	11	2	13	22	6.43E-09
	GO:0007621	BP	negative regulation of female receptivity	9	2	11	17	2.56E-08
	GO:0007320	BP	insemination	13	0	13	28	2.87E-07
	GO:0019731	BP	antibacterial humoral response	5	15	20	63	3.98E-07
	GO:0044706	BP	multi-multicellular organism process	13	0	13	29	4.77E-07
	GO:0055114	BP	oxidation-reduction process	60	50	110	764	6.04E-07
	GO:0016491	MF	oxidoreductase activity	52	50	102	679	6.18E-07
	GO:0007620	BP	copulation	13	1	14	34	6.18E-07
	GO:0045924	BP	regulation of female receptivity	11	3	14	34	6.18E-07
	GO:0017144	BP	drug metabolic process	49	23	72	443	7.68E-07
	GO:0008238	MF	exopeptidase activity	14	13	27	107	1.17E-06
	GO:0005975	BP	carbohydrate metabolic process	32	18	50	273	1.23E-06
	GO:0046692	BP	sperm competition	12	0	12	27	1.49E-06
	GO:0008235	MF	metalloexopeptidase activity	10	9	19	61	1.51E-06
	GO:0030246	MF	carbohydrate binding	21	11	32	142	1.86E-06
C vs TP6	GO:0005615	CC	extracellular space	158	36	194	749	2.90E-30
	GO:0030312	CC	external encapsulating structure	0	38	38	46	1.06E-28
	GO:0042600	CC	chorion	0	34	34	41	6.58E-26
	GO:0044421	CC	extracellular region part	168	45	213	966	4.03E-23
	GO:0005576	CC	extracellular region	196	68	264	1307	8.31E-23
	GO:0007320	BP	insemination	20	0	20	28	2.32E-13
	GO:0044706	BP	multi-multicellular organism process	20	0	20	29	6.66E-13
	GO:0046692	BP	sperm competition	19	0	19	27	1.44E-12
	GO:0007620	BP	copulation	20	1	21	34	4.46E-12
	GO:0097014	CC	ciliary plasm	27	0	27	57	1.34E-11
	GO:0005930	CC	axoneme	26	0	26	56	5.65E-11

Supplementary Table S5 (continued)

Control vs Treat	GO.ID	Domain	Term	Up	Down	DEG	Annotated	P value
	GO:0046008	BP	regulation of female receptivity, post-mating	14	1	15	22	7.11E-10
	GO:0005929	CC	cilium	46	1	47	163	9.75E-10
	GO:0044441	CC	ciliary part	36	0	36	109	1.62E-09
	GO:0032504	BP	multicellular organism reproduction	185	40	225	1378	2.14E-08
	GO:0045434	BP	negative regulation of female receptivity, post-mating	10	1	11	15	4.53E-08
	GO:0003341	BP	cilium movement	18	0	18	39	7.90E-08
	GO:0007621	BP	negative regulation of female receptivity	10	1	11	17	3.29E-07
	GO:0044447	CC	axoneme part	14	0	14	29	8.97E-07
	GO:0001539	BP	cilium or flagellum-dependent cell motility	13	0	13	25	9.31E-07
TP vs TP6	GO:0042600	CC	chorion	0	21	21	41	2.21E-19
	GO:0030312	CC	external encapsulating structure	0	21	21	46	4.76E-18
	GO:0005615	CC	extracellular space	60	5	65	749	7.13E-10
	GO:0032504	BP	multicellular organism reproduction	80	15	95	1378	3.68E-08
	GO:0007320	BP	insemination	10	0	10	28	6.26E-08
	GO:0044706	BP	multi-multicellular organism process	10	0	10	29	9.22E-08
	GO:0044421	CC	extracellular region part	62	8	70	966	2.53E-07
	GO:0005929	CC	cilium	22	0	22	163	3.58E-07
	GO:0031514	CC	motile cilium	10	0	10	34	4.22E-07
	GO:0007620	BP	copulation	10	0	10	34	5.05E-07

GO:0046692	BP	sperm competition	9	0	9	27	5.76E-07
GO:0005576	CC	extracellular region	72	14	86	1307	6.14E-07
GO:0000003	BP	reproduction	82	15	97	1515	8.83E-07
GO:0030317	BP	flagellated sperm motility	10	0	10	38	1.58E-06
GO:0097722	BP	sperm motility	10	0	10	38	1.58E-06
GO:0007286	BP	spermatid development	17	0	17	115	2.82E-06
GO:0048515	BP	spermatid differentiation	18	0	18	128	3.01E-06
GO:0036126	CC	sperm flagellum	6	0	6	13	4.66E-06
GO:0097729	CC	9+2 motile cilium	6	0	6	13	4.66E-06
GO:0097223	CC	sperm part	6	0	6	16	1.97E-05

Supplementary Table S6 List of overlapping differential metabolic markers in three groups (C vs C_H, TP vs TP_H, TP6 vs TP6_H) before and after high temperature stress in Drosophila (partial results)

NO	ID	Name	Description	Regulated
1	M203T195	3,5-Dihydroxydecanoic acid	Carbonyl compounds, production and removal in organisms is a dynamic equilibrium	up
2	M215T304_3	Undecanedioic acid	medium chain fatty acid	up
3	M187T332	Azelaic acid	Dicarboxylic acid with antibacterial and antioxidant activity	up
4	M217T345	Hydroxysebacic acid	Dicarboxylic acid, metabolised by amino acids and sugars	up
5	M87T249	Isobutyric acid	Isobutyric acid, a major metabolite of amino acids	up
6	M131T249	Ethylmalonic acid	Metabolite, functionally related to malonic acid	up
7	M173T496	Arginine	Arginine metabolism, an important amino acid metabolism that maintains normal cellular functions	up
8	M164T246_2	Phenylalanine	Phenylalanine, which plays a key role in the biosynthetic metabolism of other amino acids	up
9	M201T320	Sebacic acid	Promote, stabilise metabolism	up
10	M271T219_2	Borrelidin	Promote, stabilise metabolism	up
11	M197T51_2	12-hydroxydodecanoic acid	Active endogenous metabolites	up
12	M187T105	3-hydroxycapric acid	3-Hydroxydecanoic acid is a medium chain fatty acid	up
13	M171T53	Capric acid	Promote, stabilise metabolism	up
14	M159T131	3-hydroxyoctanoic acid	Oxidative metabolites of medium-chain fatty acids	up
15	M215T92	3-hydroxydodecanoic acid	Medium-chain fatty acids, related to fatty acid metabolism	up
16	M214T381	sn-Glycerol 3-phosphoethanolamine	Organic compounds, very strong alkaline chemistry	up
17	M258T405	Ile-Lys	dipeptide	up
18	M135T254	Threonic acid	Threonine, which plays a role in the body in balancing amino acids and regulating fat metabolism	up
19	M206T171	N-acetyl-l-phenylalanine	N-acetyl-L-phenylalanine is the product of phenylalanine N-acetyltransferase, an enzyme present in the metabolic pathway of phenylalanine	up
20	M428T63	N-oleoyl-phenylalanine	N-oleoyl phenylalanine with multiple signalling functions	up

Note: ID: metabolite ID; Name: metabolite name; Description: description of the metabolite (both from the Human Metabolome Databse and the National Library of Medicine).

Supplementary Table S7 List of metabolic markers specific to C vs TP and TP6 vs TP, respectively (partial results)

Group	ID	Name	Description	Regulated
C vs TP	M211T58	Prohexadione	Acts as a plant growth regulator and inhibitor of gibberellin biosynthesis	up
	M177T375	L-Gulonic gamma-lactone	econdary metabolites, metabolic or physiologically non-essential metabolites that may act as defence or signalling molecules	up
	M167T446	Phosphoenolpyruvate	A metabolic intermediate in pathways such as glycolysis and gluconeogenesis, with a role as a basic metabolite	up
	M341T354	Sucrose	Plays a key role in the body's stress response	up
	M401T354	Galactinol	Involved in the scavenging of reactive oxygen species and promoted the production of HSP27	up
	M563T416	Raffinose	Playing an important role in plant resistance	up
	M683T378	Cellobiose	Endogenous metabolites	up
	M133T392_2	Malate	Important organic acids produced during the metabolism of organisms	up
	M563T439	Maltotriose	A xylose metabolite	up
	M665T480	Maltotetraose	Able to break down and metabolise with high efficiency	up
TP6 vs TP	M165T112	1-methylxanthine	Primary metabolites, directly involved in the growth, development or reproduction of organisms	up
	M137T173	Salicylic acid	Activation of the plant salicylic acid signalling pathway for insect survival and reproduction	up
	M266T427	N-acetyl-d-glucosamine (N-Acetyl-D-glucosamine)	Bacterial metabolites	up
	M285T198	Hexadecanedioic acid (Hexadecanedioic acid)	Possesses anti-tumour activity	up
	M310T230	Leonurine ephedrine (Leonurus heterophyllus)	Affects homocysteine-methionine metabolism, increases methionine levels	up
	M151T208_2	Xanthine (thymine)	Purine metabolites	up
	M547T431_2	Cytidine 5'-diphosphocholine (CDP-choline)	Stabilisation and repair of biofilms, neuroprotection	up
	M243T233	Pseudouridine	Essential metabolites, a class of organic compounds called nucleosides and nucleotide analogues, end products of tRNA degradation	up
	M188T378	N-acetyl-l-glutamate (N-Acetyl-L-glutamine)	Acts as a brewer's yeast metabolite and a human metabolite	up
	M118T339_2	DL-threonine	Metabolism to produce glycine	up
